# Open Access and Scientific Societies

**DOI:** 10.1371/journal.pbio.0020156

**Published:** 2004-05-11

**Authors:** Helen Doyle, Andy Gass, Rebecca Kennison

## Abstract

Societies are encouraged to consider their own open-access experiments within the context of the communities they serve

*This is the second in a series of three editorials that aim to address recurring concerns about the benefits and risks associated with open-access publishing in medicine and the biological sciences.*

Scientific societies serve their members, their broader scholarly communities, and the different components of their missions in many important ways. Making peer-reviewed literature immediately accessible, searchable, and reusable to anyone in the world with an Internet connection is a uniquely direct means of achieving a number of goals that are common to most scholarly associations and of advancing the diverse interests of their constituencies.

Setting aside for the moment the question of how feasible it is for societies to alter their journals' access policies, there is by now a broad consensus that widespread open access to scientific publications is good for scientists and good for science. Society members want to maximize the impact of their work—and articles that are freely available online are cited more frequently than those that are not (Lawrence 2001). Most societies are committed to catalyzing innovations within and across scientific disciplines—and open-access archives of full-text literature provide a valuable tool for sharing information globally in order to accelerate the rate of scientific progress. Many societies articulate in their mission statements the goal of communicating the benefits of their members' discoveries with the public—and open-access publishing is a direct means to accomplish this goal.

In addition to an interest in exploring new ways to serve their members and their missions, societies have another compelling reason to investigate open access for their journals: the rapidly changing landscape of scholarly publishing. From 1990 to 2000, the average price of an academic journal subscription increased 10% per year (Create Change 2000). While society-run and nonprofit journals may not be the major contributors to those spiraling costs, societies that rely on revenues from subscriptions and site licenses may bear a disproportionate share of the negative consequences of skyrocketing serials prices. As libraries are forced for a variety of reasons (including decreased budgets and the increasing prevalence of “big deals” and journal bundling) to eliminate subscriptions, society journals may be among the hardest hit. Journals that appeal to a relatively specialized readership and those that are not part of larger publishing groups are particularly vulnerable to the contraction of serials collections that has already begun and will likely accelerate (Create Change 2000).

## A Society Is More Than a Journal

The confluence of forces in favor of open access says nothing about its fiscal implications for scientific societies. As any systemic change in research or publishing would, the movement toward open access has generated concern about its ramifications for the scholarly associations that often serve as the backbones of scientific communities. However, the strength of those societies and their essential role in the communities they serve are precisely what should allay fears about the revenue-eroding effect that some argue would plague societies if they converted their traditional subscription-based journals to open access.

Scientific societies perform an array of tremendously valuable functions for their constituents and disciplines. Researchers, educators, and others join societies for the many benefits of membership beyond simply discounted or “free” subscriptions to journals, so the concern that open-access publications would be the death knell of voluntary academic associations is misguided. As Elizabeth Marincola, executive director of the American Society for Cell Biology, recently noted, her society “offers a diverse range of products so that if publications were at risk financially, we wouldn't lose our membership base because there are lots of other reasons why people are members” (Anonymous 2003).

While open-access publication can, in fact, be paid for in a number of different ways, there is no question that a transition toward the elimination of online access barriers requires most societies to restructure the business models for their journals. If journal subscriptions generate surplus revenue that supports other society activities, then the business model of the society as a whole may need to be examined. This is not to say that open-access journals cannot generate a surplus or profit—simply that they do not do so by restricting access to their primary research content.

## Testing the Open-Access Waters

There are a number of societies that have already begun to take transitional steps to wean themselves from subscription revenues. One of the earliest societies to commit to open-access publication, the American Society for Clinical Investigation (ASCI) has since 1996 provided the *Journal of Clinical Investigation (JCI)* freely online and recently reaffirmed its commitment to open access: “The financing having been resolved, through author charges and other means,” John Hawley, the executive director of the ASCI writes, “the *JCI* hopefully can bring the greatest benefit to its authors and readers, regardless of who they might be. It is in this spirit that the *JCI* has always been free online, and will remain so” (Hawley 2003).

In order to experiment cautiously with new access policies, several societies have implemented hybrid models of access-restriction for their publications. The American Physiological Society, for example, offers authors in *Physiological Genomics* the option to pay a surcharge for their articles to be made freely available online immediately upon publication. A recent survey by the Joint Information Systems Committee (JISC) in the United Kingdom suggests that many authors would use such an option if it were more widely available: 48% of authors who had never published in an open-access journal and 60% of authors who had done so indicated that they would be willing to “pay a publisher of a journal sold according to the traditional subscription model an additional fee for them to make [the author's] particular paper ‘open access’” (JISC 2004).

JISC is also directly encouraging society and nonprofit publishers to implement hybrid models and other open-access experiments and to launch new open-access journals by providing grants to offset the publication charges for authors during this transitional phase. In the long run, of course, open access will prove sustainable when more funders of research, in addition to interested third parties, designate funds specifically for the costs of publishing articles to be made freely available, searchable, and reusable online.

## Starting the Dialogue

Reaching a “steady-state” system of open-access publishing by scientific societies will require three critical components: recognition that open access serves societies' members and missions; diversified revenue streams not solely dependent on subscription or site-license fees; and society publishers' making use of recent innovations in journal production and dissemination, which can dramatically reduce the costs of publishing. It is, after all, the increased efficiencies born of new technologies—from the Internet itself to electronic journal management systems—that have made the idea of open access possible. And while proponents of open access are confident that publication charges of around $1,500 per article will be sufficient to cover the costs of publishing an efficiently operated society journal, there is no question that many existing journals may need to update their infrastructure in order to make open access financially viable (PLoS 2004).

There is also no question that many societies do not, at present, have a wealth of revenue streams beyond the proceeds from their journals, which they often use to fund valuable activities from education initiatives to annual meetings. As open-access journals become more established, however, and as the benefits of open access to scientific and medical literature become more apparent to society members, the demand for the broadest possible dissemination of research is only likely to grow. Those societies that embrace the developments taking place in scholarly publishing may well see their membership and publications thrive more than societies that cling to the potentially unstable status quo.

In any case, a constructive discussion about the pitfalls to be avoided and the benefits to be gained through a transition to open-access publishing would be a worthy first step for any scientific society to take—and PLoS welcomes the questions, comments, and feedback of those who are intrigued by the potential that open access affords and want to learn more.
